# Impact of the neutral position and rotation of the head in ultrasound-guided internal jugular vein catheterization on duration of procedure and complications

**DOI:** 10.1186/cc13315

**Published:** 2014-03-17

**Authors:** M Kurt, A Ozgultekin, G Turan, F Ormanci, S Batan, O Ekinci

**Affiliations:** 1 Haydarpasa Numune Teaching and Research Hospital, Istanbul, Turkey

## Introduction

In internal jugular vein (IJV) catheterization, neck rotation may enhance the visibility of anatomical landmarks; however, it may increase the risk of carotid artery (CA) puncture by replacing the position of the IJV in relation to the CA. In our study, during US- guided IJV catheterizations, we investigated the effects of changing the position of the IJV by the CA depending on the neutral position and 45° rotation of the head on duration of procedure and complications.

## Methods

After obtaining hospital ethics committee approval, 100 intensive care patients aged >18 years were included in the study. Patients were randomly selected and catheterization was performed in a neutral position of the head (*n *= 50) and by turning the head 45° to the opposite side (*n *= 50). The US-guided catheterization procedure was performed in accordance with general principles. Once the needle entered the IJV and blood was aspirated, the US probe was released from the hand, and the catheter was placed and fixed according to the Seldinger technique. The data of the intervention side for each process, the count number of successful catheter insertion, whether there is arterial access or not and the duration of procedure (from skin contact of the needle to catheter insertion) were recorded.

## Results

The localization of the IJV in relation to the CA is 66% anterolateral, 4% anterior and 30% lateral in a neutral position, and 62% anterolateral, 28% anterior, 10% lateral position in 45° rotation. So while there is no change in a significant proportion of patients in the localization of the IJV in relation to the CA, the anterior placement rate increasing the risk of CA puncture was significantly higher in a position of rotation compared with the neutral position (*P *= 0.001). No significant difference was found between procedure durations. Complications were recorded when observed.

## Conclusion

In our study, it has been shown that anterior placement of the IJV in the neutral position is less but this has no advantage in avoiding arterial puncture. The smaller area of procedure in a neutral position can cause difficulties in practice. However, the processing times between each head position were not different. Nevertheless, further studies evaluating whether there are comparable complication rates and the same duration of procedure in emergency and trauma patients in whom head rotation cannot be possible are needed.

